# Diagnostic Performance of Deep Learning Classifiers in Measuring Peripheral Anterior Synechia Based on Swept Source Optical Coherence Tomography Images

**DOI:** 10.3389/fmed.2021.775711

**Published:** 2022-01-26

**Authors:** Yangfan Yang, Yanyan Wu, Chong Guo, Ying Han, Mingjie Deng, Haotian Lin, Minbin Yu

**Affiliations:** ^1^State Key Laboratory of Ophthalmology, Zhongshan Ophthalmic Center, Sun Yat-sen University, Guangzhou, China; ^2^Department of Ophthalmology, University of California, San Francisco, San Francisco, CA, United States

**Keywords:** anterior chamber angle, deep learning, primary angle closure disease, artificial intelligence, swept source optical coherence tomography

## Abstract

**Purpose:**

To develop deep learning classifiers and evaluate their diagnostic performance in detecting the static gonioscopic angle closure and peripheral anterior synechia (PAS) based on swept source optical coherence tomography (SS-OCT) images.

**Materials and Methods:**

Subjects were recruited from the Glaucoma Service at Zhongshan Ophthalmic Center of Sun Yat-sun University, Guangzhou, China. Each subject underwent a complete ocular examination, such as gonioscopy and SS-OCT imaging. Two deep learning classifiers, using convolutional neural networks (CNNs), were developed to diagnose the static gonioscopic angle closure and to differentiate appositional from synechial angle closure based on SS-OCT images. Area under the receiver operating characteristic (ROC) curve (AUC) was used as outcome measure to evaluate the diagnostic performance of two deep learning systems.

**Results:**

A total of 439 eyes of 278 Chinese patients, which contained 175 eyes of positive PAS, were recruited to develop diagnostic models. For the diagnosis of static gonioscopic angle closure, the first deep learning classifier achieved an AUC of 0.963 (95% *CI*, 0.954–0.972) with a sensitivity of 0.929 and a specificity of 0.877. The AUC of the second deep learning classifier distinguishing appositional from synechial angle closure was 0.873 (95% *CI*, 0.864–0.882) with a sensitivity of 0.846 and a specificity of 0.764.

**Conclusion:**

Deep learning systems based on SS-OCT images showed good diagnostic performance for gonioscopic angle closure and moderate performance in the detection of PAS.

## Introduction

Glaucoma is the main cause of irreversible blindness (1), affecting an estimated 76 million people worldwide (2). In Asia, the prevalence of primary angle closure disease (PACD) is expected to increase significantly to reach 34 million in 2040 (2). PACD has chronic and acute forms, which may lead to severe eye pain and rapid loss of vision, or irreversible blindness if untreated (3).

Early intervention and treatment of PACD depend on early detection, which requires assessment of the anterior chamber angle (ACA). Gonioscopy is a gold standard for assessing the ACA configuration and detecting PAS in clinics. However, it is a contact examination that should not be used on some patients due to safety concerns.

Swept source optical coherence tomography (SS-OCT) is a non-contact imaging technique providing high resolution images of anterior segment structures. Compared to traditional anterior segment optical coherence tomography (AS-OCT), the laser wavelength of SS-OCT is 1,310 nm, which enables it to scan and store 360-degree images of the anterior chamber in a few seconds with high reproducibility and definition (3). Previous studies have shown that SS-OCT exhibits moderate performance for angle closure detection compared with gonioscopy as the reference standard (4), although not all studies were in agreement (5). A limitation of AS-OCT for angle closure detection is its limited ability to distinguish appositional angle closure and synechial angle closure based on two-dimensional cross-sectional images. However, a previous study by Leung et al. had indicated that it was feasible to discriminate synechial angle closure from the appositional angle closure with dynamic paired dark-light AS-OCT imaging, which showed that synechial closure often exhibited an obtuse configuration while appositional closure assumed an acute configuration (6). This shows the possibility to explore the way of discriminating these two kinds of angle closure based on static SS-OCT imaging.

Deep learning, a branch of machine learning, has been used to detect diabetic retinopathy, age-related macular degeneration, retinopathy of prematurity, glaucoma, and cataract by learning images from fundus photographs, perimetry, fundus OCT, and slit lamp microscopy (7–12). Recent studies have suggested that AS-OCT combined with deep learning has the potential to detect narrow ACAs in at-risk people (13, 14), indicating that it is possible to develop a deep learning system based on SS-OCT images to detect the appositional angle closure.

The purpose of this study was to develop an artificial intelligence system for ACA detection based on SS-OCT images. To achieve this aim, two deep learning classifiers were developed, the first to diagnose static gonioscopic angle closure and the second to distinguish appositional angle closure from synechial angle closure.

## Materials and Methods

This prospective observational study adhered to the tenets of the Declaration of Helsinki and was approved by the Institutional Review Boards of Zhongshan Ophthalmic Center, Guangzhou, China (2018KYPJ007) and registered at ClinicalTrials.gov (NCT03611387). Informed consent was obtained from all patients.

A total of 278 participants of the Zhongshan Ophthalmic Center were enrolled in this study between August 2018 and August 2020. Patients eligible for inclusion were phakic, over 18 years old, and able to undergo both gonioscopy and SS-OCT examinations. Exclusion criteria included the history of prior intraocular surgery and laser treatment, prior history of APAC, acute ocular inflammation, secondary angle closure, open angle glaucoma, or corneal opacities that disturbed the visualization and imaging of ACA. Both eyes of a single participant were included if they met the above criteria.

### Clinical Evaluation

All eyes included in this study underwent a series of standard ophthalmic examinations, such as ACA evaluation by gonioscopy and SS-OCT imaging. Gonioscopy was performed by six glaucoma experts using a 1-mirror gonioscope (Volk Optical Inc, Mentor, OH, USA) and a 1-mm light beam placed off the pupil to avoid pupillary constriction. If the pigmented trabecular meshwork could not be visualized by static examination, dynamic gonioscopy was performed to evaluate the presence of PAS. The gonioscopy result combined static and dynamic examination, and the range of PAS was recorded based on clock position which was also converted into degree position.

Swept source optical coherence tomography (SS-OCT) (CASIA SS-1000, Tomey Corporation, Nagoya, Japan) imaging was performed in a dark room by a trained examiner masked to gonioscopic findings. During the scanning, the upper eyelids were gently retracted to decrease superior eyelid artifacts, while taking care to avoid inadvertent pressure on the globe. Three-dimensional angle analysis mode was used with 512 A-scans per line which takes 128 2-dimensional cross-sectional images per eye.

### SS-OCT Images Dataset

**Figure 1:**
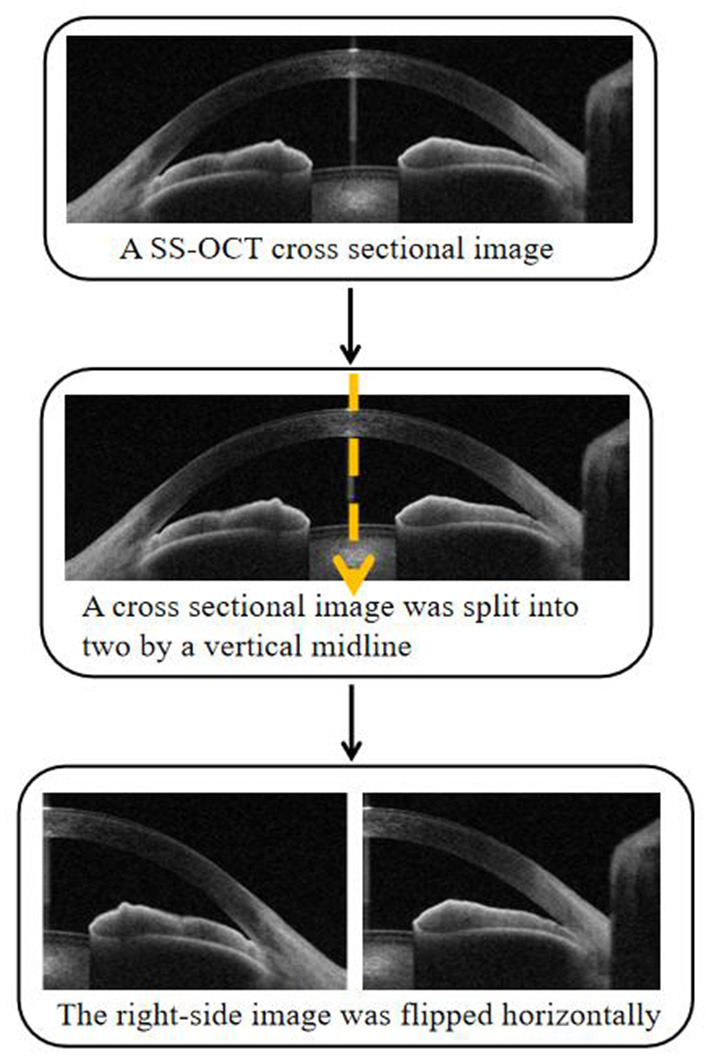
Each swept source optical coherence tomography (SS-OCT) cross-sectional image was split into two anterior chamber angle (ACA) images with the right-side image flipped horizontally.

The SS-OCT Viewer software (version 4.7; Tomey Corporation, Nagoya, Japan) was used to export 128 cross-sectional images in JPEG format from raw image data per eye. Each SS-OCT cross-sectional image was split into two by a vertical midline with the right-side image flipped horizontally to match the orientation of the left-side image. Images with motion artifacts and incomplete images were excluded from the analysis (Figure 1).

Each ACA image was classified by two trained observers as an open-angle or static angle closure. In case of disagreement, a senior glaucoma specialist adjudicated. The static angle closure in the SS-OCT ACA image was defined as a substantial iris-angle contact beyond the scleral spur while an open angle in the ACA image was defined as no iridotrabecular contact anterior to the scleral spur. This procedure yielded the first SS-OCT dataset containing ACA images with the open angle and static angle closure images.

**Figure 2:**
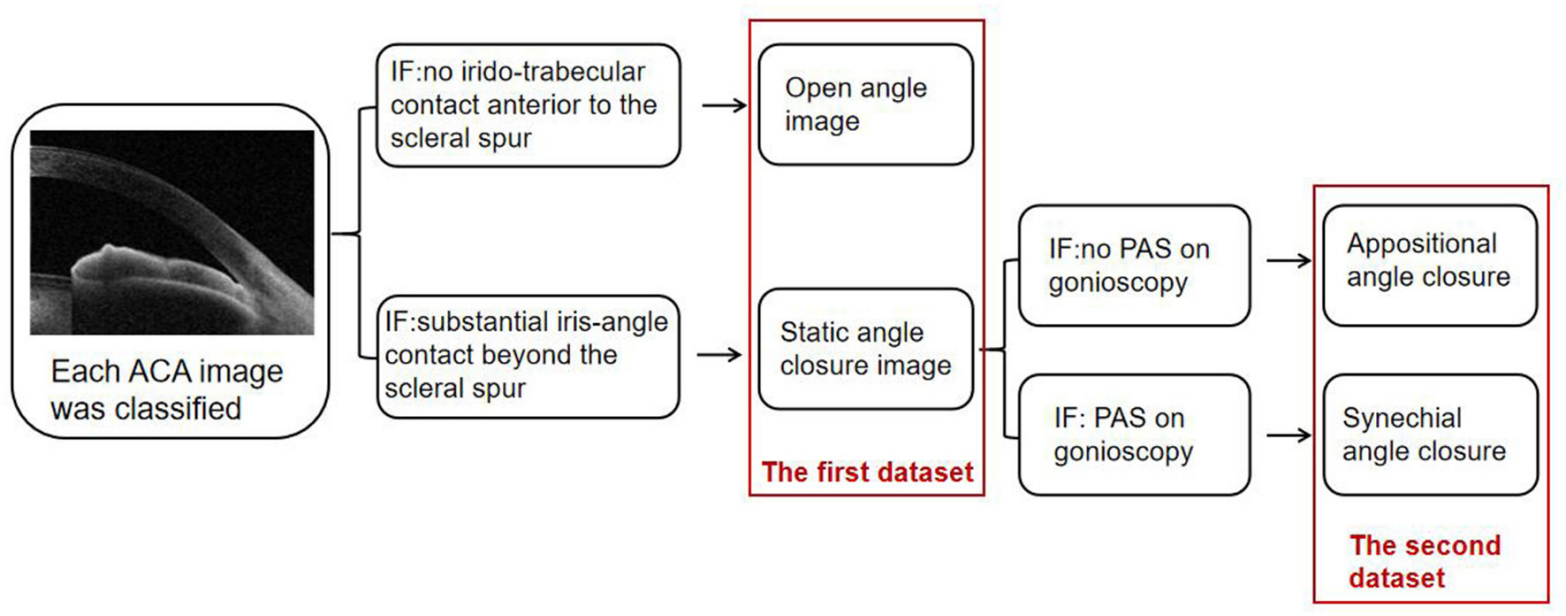
Each ACA image was first classified into an open angle or static angle closure. Then ACA images of the static angle closure were combined with gonioscopy to be reclassified into appositional angle closure or synechial angle closure.

The static angle closure ACA images in the first SS-OCT dataset were combined with gonioscopy results to reclassify those ACA images as appositional angle closure or synechial angle closure. Appositional angle closure in the static angle closure ACA images was defined as no PAS recorded on gonioscopy. Synechial angle closure images were defined as PAS recorded on gonioscopy. This procedure yielded the second SS-OCT dataset. ACA images corresponded to within 15 degrees before and after the starting and ending points of PAS were excluded. The image handling process is shown schematically in Figure 2.

Image pixel values were normalized to range between 0 and 1. Images were randomly rotated between 0 and 15 degrees and randomly shifted horizontally and vertically as means of images augmentation (15), to enhance the robustness of the model during classifier training.

### Deep Learning System

**Figure 3:**
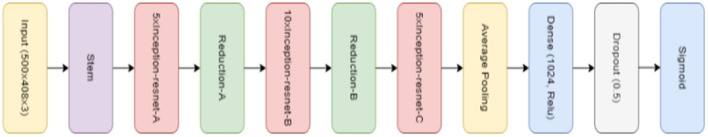
The thumbnail view of the entire convolutional neural network (CNN) architecture.

Two deep learning classifiers were developed in this study using a convolutional neural network (CNN) approach. Both deep learning classifiers were based on the InceptionResnetV2 CNN architecture consisting of 244 convolution layers, 204 batch normalization layers, and four pooling layers. The fully connected layer with a size of 1,024 linked to a dropout layer with a retention rate of 0.5 predicted a binary classification result with a sigmoid activation function and a binary cross-entropy loss function in the final output layer. The adaptive moment estimation optimizer with an initial learning rate of 0.001, beta 1 of 0.9, beta 2 of 0.999, fuzzy coefficient of 1e-7, and learning decay rate of 0 were applied. The thumbnail view of the entire CNN architecture is shown in Figure 3.

The CNN architecture initialization was performed by the pre-trained model obtained from ImageNet classification (16). Each model was trained up to 500 epochs. After each epoch, the validation loss was evaluated using the validation set and used as a reference for model selection. The training process was stopped if the validation loss did not improve over 120 consecutive epochs. The model with the lowest validation loss was saved as the final model.

All ACA images of eyes included in the first SS-OCT dataset were randomly segregated into three sub-sets, with 70% of images for training, 15% of images for validation, and 15% of images as a test dataset, to develop the first deep learning classifier. Images in each dataset were independent without crossover or overlap between datasets. The second deep learning classifier was developed by randomly allocating ACA images relabeled in the second SS-OCT dataset into three sub-sets, with 80% of images as a training set, 10% of images as a validation set, and 10% of images as a test set.

### Test Classifier

**Figure 4:**
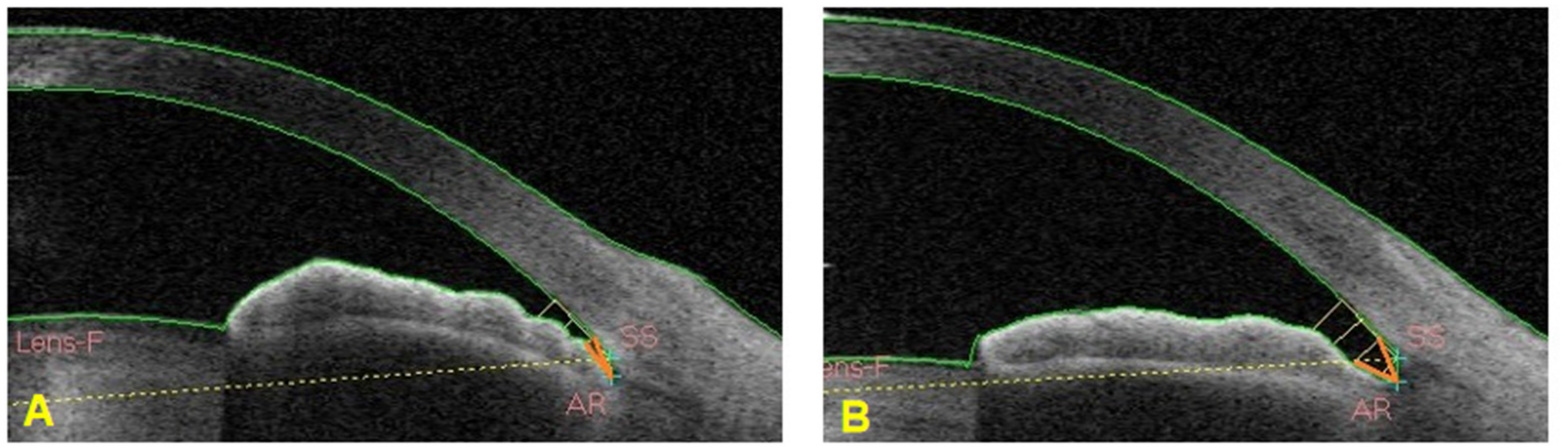
TIA250 was marked by an orange angle. **(A)** The ACA image showed TIA250 between 11 and 15 degrees. **(B)** The ACA image showed TIA250 >25 degrees.

At the early stage of this study, a test classifier was developed that included a small sample and several ACA images. To test the accuracy of the classifier in distinguishing different widths of an open angle, we selected two different and independent test sets. Open angle images in the test set 1 were images with wider trabecular iris angle 250 um (TIA250) while open angle images in test set 2 were images with narrower TIA250. Images with TIA250 >25 degrees were selected in the test set 1 while images in the test set 2 were those with TIA250 ranging from 11 to 15 degrees (Figure 4).

### Statistical Analysis

Model performance was assessed using three critical outcome measures: accuracy, sensitivity, and specificity. A receiver operating characteristic (ROC) curve was used to evaluate the efficacy of deep learning classifiers. The classification threshold was measured by the Youden index (17). Moreover, the area under the ROC curve (AUC) with 95% CI was chosen to evaluate the deep learning models.

## Result

A total of 77,613 ACA images from 439 eyes were captured that concluded 165 eyes of normal, 99 eyes of primary angle closure suspect (PACS), 85 eyes of primary angle closure (PAC), and 90 eyes of primary angle closure glaucoma (PACG). The participant's demographics are shown in Table 1.

**Table 1:** Participants demographics.

	**Normal eyes**	**PACS eyes**	**PAC eyes**	**PACG eyes**
Number	164	112	80	83
Age (years)	59.37 ± 16.46	62.45 ± 7.94	59.75 ± 10.40	59.58 ± 12.60
Males (%)	76 (46.3%)	30 (26.8%)	27 (33.8%)	29 (34.9%)
Females (%)	88 (53.7%)	82 (73.2%)	53 (66.2%)	54 (65.1%)

**Figure 5:**
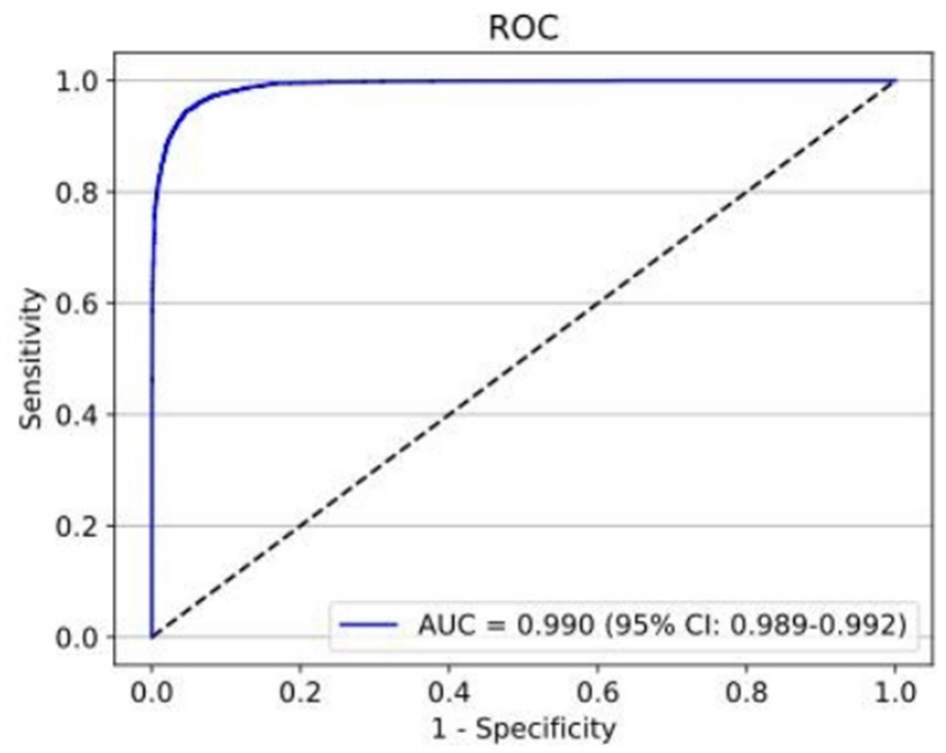
The test set performance for the detection of static angle closure and open angle achieved an AUC of 0.990 with a sensitivity of 0.9465 and specificity of 0.9533.

The first deep learning classifier was developed with 50,650 ACA images as a training set (34,705 open angle images and 15,945 static angle closure images), 11,291 ACA images as a validation set (8,037 open angle and 3,254 static angle closure images), and 10,884 ACA images as a test set (7,860 open angle and 3,024 static angle closure images). To distinguish the static angle closure and open angle based on ACA images, the first deep learning classifier achieved an AUC of 0.990 (95% *CI:* 0.989–0.992) with a sensitivity of 0.9465 and specificity of 0.9533 (Figure 5).

**Figure 6:**
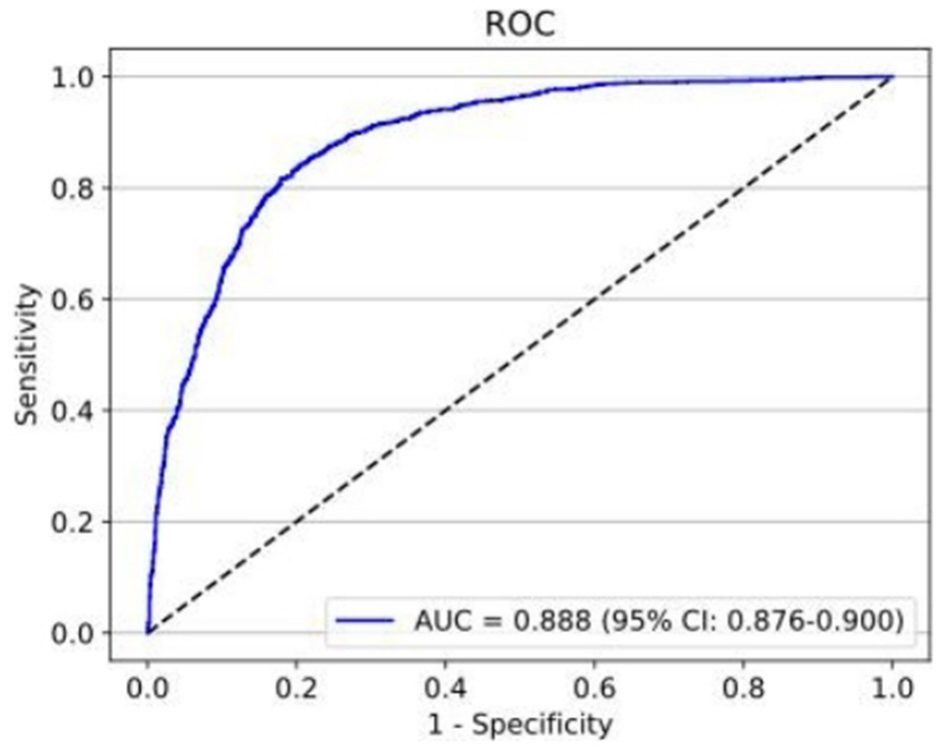
The test set performance for the detection of PAS achieved an AUC of 0.888 with 82.7% sensitivity and 80.7% specificity.

The second deep learning classifier contained the training set with 21,924 ACA images (10,140 appositional angle closure images and 11,784 synechial angle closure images), validation set with 2,612 images (1,399 appositional angle closure images and 1,213 synechial angle closure images), and test set with 2,475 images (1,282 appositional angle closure images and 1,193 synechial angle closure images). The performance of the second deep learning classifier for detecting PAS achieved an AUC of 0.888 (95% *CI:* 0.876–0.900) with 82.7% sensitivity and 80.7% specificity (Figure 6).

**Figure 7:**
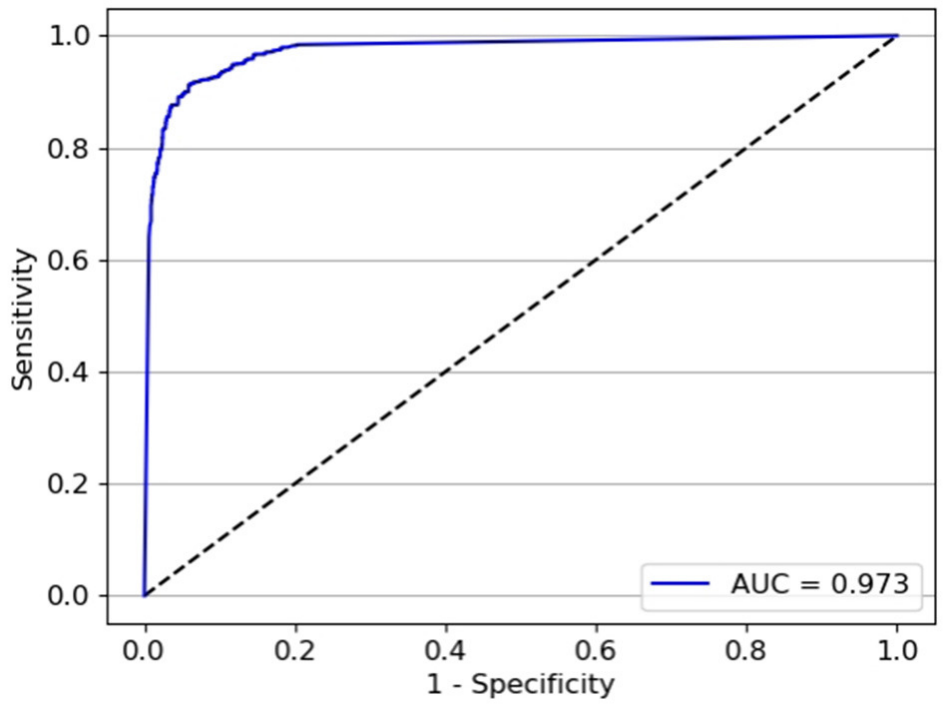
The performance of test set 1 for the test classifier achieved an AUC of 0.973 with a sensitivity of 93.4% and specificity of 94.6%.

**Figure 8:**
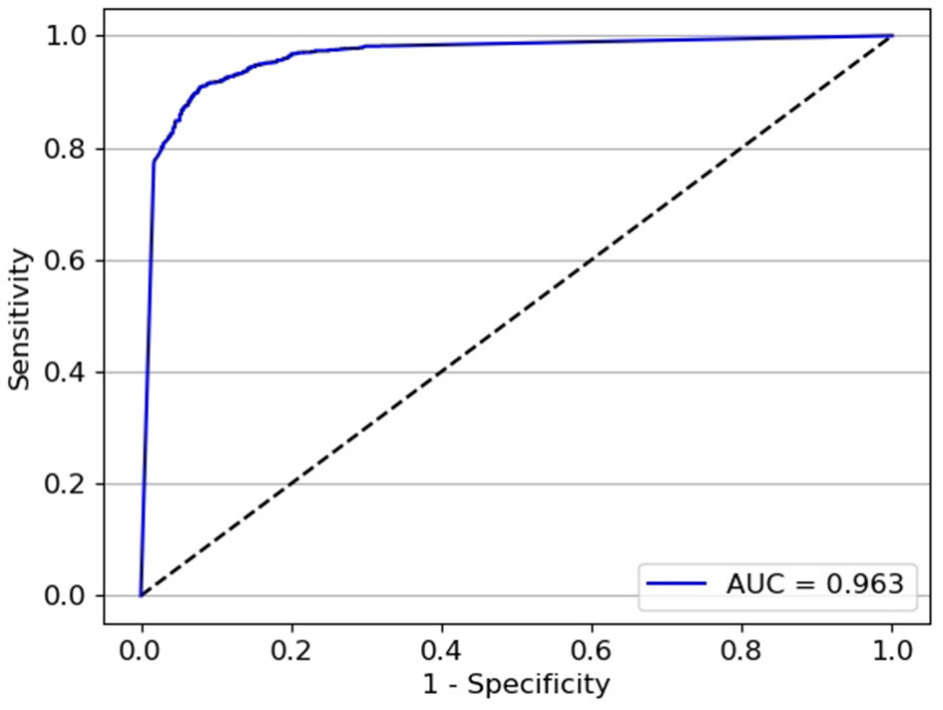
The performance of test set 2 for the test classifier achieved an AUC of 0.963 with a sensitivity of 92.9% and specificity of 87.7%.

The test classifier was developed with 20,382 ACA images (7,798 open angle and 12,785 static angle closure) as the training set, and 4,320 ACA images (2,160 open angle and 2,160 static angle closure) as the validation set. The test set 1 contained 2,866 ACA images with 1,807 open angle and 1,059 static angle closure images; test set 2 contained 3,035 ACA images with 1,218 open angle and 1,817 static angle closure images. The AUCs for the test classifier detection of different widths of open angle were 0.973 (95% *CI:* 0.972–0.975) in the test set 1 (constructed by images with wider TIA250) and 0.963 (95% *CI:* 0.960–0.965) in the test set 2 (constructed by images with narrower TIA250). The test set 1 showed a sensitivity of 93.4% and specificity of 94.6% (Figure 7), while the test set 2 showed a sensitivity of 92.9% and specificity of 87.7% (Figure 8).

## Discussion

Medical artificial intelligence has been successfully applied to the breast cancer diagnosis and classification of skin cancer (18, 19). With an impressive learning ability and accuracy in the interpretation of images, natural languages, and speech processing (20), deep learning has been widely used for medical image-based artificial intelligence (AI) research. Glaucoma AI detection mainly utilizes color fundus photographs, fundus OCT, visual field, and AS-OCT alone or together (10, 11). As is well-known, if acute PACG is not treated in a timely fashion, it can rapidly cause irreversible b*l*indness (21). Therefore, the AI detection of PACD should be focused on early detection as a basis for treatment.

In this study, we successfully established a deep learning system to identify the ACA status in terms of open angle, appositional angle closure, and synechial angle closure based on SS-OCT images. In contrast to the previous research on ACA AI detection (13, 14), we focused on high accuracy detection of static angle closure based on SS-COT images.

Previous studies have used AS-OCT for angle closure diagnosis. Fu et al. developed two angle closure detection systems, one of which was a quantitative feature-based support vector machine classifier and another was a deep learning classifier with a VGG-16 network, based on a total of 41,35 Visante AS-OCT images (13). The former achieved an AUC of 0.90 with a sensitivity of 79% and a specificity of 87% while the latter achieved an AUC of 0.96 with a sensitivity of 0.90 and a specificity of 0.92, demonstrating better detection accuracy with deep learning than with the linear support vector machine classifier. Xu et al. tested multiclass convolutional network classifiers to automatically detect the angle closure based on 4036 SS-OCT images (14) and found that three CNN classifiers showed excellent performance with AUC from 0.90 to 0.93. Recently, an Angle Closure Evaluation Challenge provided a dataset containing 4,800 AS-OCT images for several teams to develop AI classifiers (22). Nine teams established different deep learning models for angle closure detection which achieved AUCs from 0.9 to 0.96 with sensitivities between 0.79 and 0.94 and specificities from 0.87 to 0.91.

The InceptionResnetV2 network, which achieved the highest accuracy of image classification in the ImageNet Large Scale Visual Recognition Challenge (16), is a modified InceptionV3 network and develops deeper neural networks by importing the residual connection to obtain better performance. In the present study, a test classifier based on the InceptionResnetV2 network was developed to discriminate different widths of open angle and closed angle. In discrimination between static angle closure and open angle with wider TIA250, this classifier achieved an AUC of 0.973, and a slightly lower AUC of 0.963 was achieved for discrimination between static angle closure detection and open angle with narrower TIA250. This performance indicates that the differentiation between open angle with narrower TIA250 and static angle closure is more difficult than between open with wider TIA250 and closed angles, but both showed that the network does work in different situations. The training dataset was then increased to develop the first deep learning classifier to discern open angle from the static angle closure, the result of which was a very high AUC of 0.990. Therefore, deep learning achieved reliable performance in detecting the static angle closure based on AS-OCT images but requires large sample sizes and deeper training to improve its capability.

Although deep learning systems have helped to reliably detect the static angle closure, it remains difficult to distinguish the appositional angle closure from the synechial angle closure based on static AS-OCT images. Therefore, the second deep learning classifier based on the SS-OCT dataset combined with dynamic gonioscopy as a reference standard was trained to distinguish between the appositional and synechial angle closure and achieved a moderate AUC of 0.888. Recently, another study focused on classifying the appositional and synechial angle closure. Hao et al. developed five traditional machine classifiers and 13 CNN deep learning models based on 8,848 AS-OCT images collected in bright and dark environments (23). In their study, the best result of traditional machine classifiers was an AUC of 0.64, while all CNN deep learning models achieved higher AUCs of up to 0.84. The results of Hao's et al. (23) research and the second deep learning classifier in our study show that deep learning holds potential in measuring PAS based on SS-OCT images. Additional datasets and model training are required to develop a deep learning system to reliably distinguish between the appositional and synechial angle closure. In addition, we found that all heatmaps of these two deep learning classifiers focused on the ACA (as figures showed in Supplementary Materials), which were similar to previous studies and supported that iridocorneal junction provided highly discriminative structure features on the angle closure detection (13, 14). However, we still could not identify the precise features between the appositional angle closure and synechial angle closure from heatmaps. So, further study is needed to learn what enables deep learning classifiers to distinguish the different statuses of ACA.

Our study has some limitations. The performance of the two classifiers was within a research environment and may differ when tested using real world datasets. Another limitation of our study is that it included a Chinese sample, and the results may not apply to the other population settings. Therefore, a multi-center study with larger datasets and multiple devices is needed to evaluate the generalizability and accuracy of the deep learning system for ACA status detection.

In conclusion, we developed two deep learning classifiers based on SS-OCT images, which showed excellent performance in distinguishing gonioscopic angle closure from open angles but moderate performance on the detection of peripheral anterior synechiae. SS-OCT holds potential in the evaluation of precise angle structure status so that it may be used for screening high-risk glaucoma populations in the future.

## Data Availability Statement

The original contributions presented in the study are included in the article/Supplementary Material, further inquiries can be directed to the corresponding author/s.

## Ethics Statement

The studies involving human participants were reviewed and approved by Institutional Review Boards of Zhongshan Ophthalmic Center, Guangzhou, China. The patients/participants provided their written informed consent to participate in this study.

## Author Contributions

YY, YW, HL, and MY contributed to the conception and design. YY and YW contributed to the drafting and refining of the manuscript. HL and MY performed the critical reading of the manuscript. All authors have read and approved the manuscript.

## Funding

This work was supported by grants from the National Natural Science Foundation of China (Grant No. 81970847) and the Natural Science Foundation of Guangdong Province (Grant No. 2021A1515010604).

## Conflict of Interest

The authors declare that the research was conducted in the absence of any commercial or financial relationships that could be construed as a potential conflict of interest.

## Publisher's Note

All claims expressed in this article are solely those of the authors and do not necessarily represent those of their affiliated organizations, or those of the publisher, the editors and the reviewers. Any product that may be evaluated in this article, or claim that may be made by its manufacturer, is not guaranteed or endorsed by the publisher.

## References

[1] Bourne RRA Flaxman SR Braithwaite T Cicinelli MV Das A Jonas JB. Magnitude, temporal trends, and projections of the global prevalence of blindness and distance and near vision impairment: a systematic review and meta-analysis. Lancet Glob Health. (2017) 5:e888–97. 10.1016/S2214-109X(17)30293-0 28779882

[2] Tham YC Li X Wong TY Quigley HA Aung T Cheng CY. Global prevalence of glaucoma and projections of glaucoma burden through 2040: a systematic review and meta-analysis. Ophthalmology. (2014) 121:2081–90. 10.1016/j.ophtha.2014.05.013 24974815

[3] Chansangpetch S Rojanapongpun P Lin SC. Anterior segment imaging for angle closure. Am J Ophthalmol. (2018) 188:xvi–ix. 10.1016/j.ajo.2018.01.006 29352976

[4] Porporato N Baskaran M Tun TA Sultana R Tan MCL Quah JHM. Assessment of circumferential angle closure with swept-source optical coherence tomography: a community based study. Am J Ophthalmol. (2019) 199:133–9. 10.1016/j.ajo.2018.11.015 30502338

[5] Nolan WP See JL Chew PT Friedman DS Smith SD Radhakrishnan S. Detection of primary angle closure using anterior segment optical coherence tomography in Asian eyes. Ophthalmology. (2007) 114:33–9. 10.1016/j.ophtha.2006.05.073 17070597

[6] Lai I Mak H Lai G Yu M Lam DS Leung CK. Anterior chamber angle imaging with swept-source optical coherence tomography: measuring peripheral anterior synechia in glaucoma. Ophthalmology. (2013) 120:1144–9. 10.1016/j.ophtha.2012.12.006 23522970

[7] Gulshan V Peng L Coram M Stumpe MC Wu D Narayanaswamy A. Development and validation of a deep learning algorithm for detection of diabetic retinopathy in retinal fundus photographs. JAMA. (2016) 316:2402–10. 10.1001/jama.2016.17216 27898976

[8] Burlina PM Joshi N Pekala M Pacheco KD Freund DE Bressler NM. Automated grading of age-related macular degeneration from color fundus images using deep convolutional neural networks. JAMA Ophthalmol. (2017) 135:1170–6. 10.1001/jamaophthalmol.2017.3782 28973096 PMC5710387

[9] Brown JM Campbell JP Beers A Chang K Ostmo S Chan RVP. Automated diagnosis of plus disease in retinopathy of prematurity using deep convolutional neural networks. JAMA Ophthalmol. (2018) 136:803–10. 10.1001/jamaophthalmol.2018.1934 29801159 PMC6136045

[10] Halupka KJ Antony BJ Lee MH Lucy KA Rai RS Ishikawa H. Retinal optical coherence tomography image enhancement via deep learning. Biomed Optics Express. (2018) 9:6205–21. 10.1364/BOE.9.006205 31065423 PMC6490980

[11] Elze T Pasquale LR Shen LQ Chen TC Wiggs JL Bex PJ. Patterns of functional vision loss in glaucoma determined with archetypal analysis. J R Soc Interface. (2015) 12:20141118. 10.1098/rsif.2014.1118 25505132 PMC4305414

[12] Long E Lin H Liu Z Wu X Wang L Jiang J. An artificial intelligence platform for the multihospital collaborative management of congenital cataracts. Nat Biomed Eng. (2017) 1:0024. 10.1038/s41551-016-0024

[13] Fu H Baskaran M Xu Y Lin S Wong DWK Liu J. A deep learning system for automated angle-closure detection in anterior segment optical coherence tomography images. Am J Ophthalmol. (2019) 203:37–45. 10.1016/j.ajo.2019.02.028 30849350

[14] Xu BY Chiang M Chaudhary S Kulkarni S Pardeshi AA Varma R. Deep learning classifiers for automated detection of gonioscopic angle closure based on anterior segment OCT images. Am J Ophthalmol. (2019) 208:273–80. 10.1016/j.ajo.2019.08.004 31445003 PMC6888901

[15] Krizhevsky A Sutskever I Hinton GE. ImageNet classification with deep convolutional neural networks. J Commun. (2017) 60:84–90. 10.1145/3065386

[16] Russakovsky O Deng J Su H Krause J Satheesh S Ma S. ImageNet large scale visual recognition challenge. Int J Comp Vis. (2015) 115:211–52. 10.1007/s11263-015-0816-y

[17] Yu M Tham YC Rim TH Ting DSW Wong TY Cheng CY. Reporting on deep learning algorithms in health care. Lancet Digit Health. (2019) 1:e328–9. 10.1016/S2589-7500(19)30132-3 33323206

[18] Esteva A Kuprel B Novoa RA Ko J Swetter SM Blau HM. Dermatologist-level classification of skin cancer with deep neural networks. Nature. (2017) 542:115–8. 10.1038/nature21056 28117445 PMC8382232

[19] Somashekhar SP Sepúlveda MJ Puglielli S Norden AD Shortliffe EH Rohit Kumar C. Watson for oncology and breast cancer treatment recommendations: agreement with an expert multidisciplinary tumor board. Ann Oncol. (2018) 29:418–23. 10.1093/annonc/mdx781 29324970

[20] Esteva A Robicquet A Ramsundar B Kuleshov V DePristo M Chou K. A guide to deep learning in healthcare. Nat Med. (2019) 25:24–9. 10.1038/s41591-018-0316-z 30617335

[21] Stein JD Khawaja AP Weizer JS. Glaucoma in adults-screening, diagnosis, and management: a review. JAMA. (2021) 325:164–74. 10.1001/jama.2020.21899 33433580

[22] Fu H Li F Sun X Cao X Liao J Orlando JI. AGE challenge: angle closure glaucoma evaluation in anterior segment optical coherence tomography. Med Image Anal. (2020) 66:101798. 10.1016/j.media.2020.101798 32896781

[23] Hao H Zhao Y Yan Q Higashita R Zhang J Zhao Y. Angle-closure assessment in anterior segment OCT images via deep learning. Med Image Anal. (2021) 69:101956. 10.1016/j.media.2021.101956 33550010

