# Supplementary material for: Diagnostic Performance of Deep Learning Classifiers in Measuring Peripheral Anterior Synechia Based on Swept Source Optical Coherence Tomography Images
Source: Front Med (Lausanne). 2022 Jan 26;8:775711. doi: 10.3389/fmed.2021.775711 (PMC8825342; doi:10.3389/fmed.2021.775711)
Supplement: Supplementary file 2 [file Data_Sheet_1.PDF]

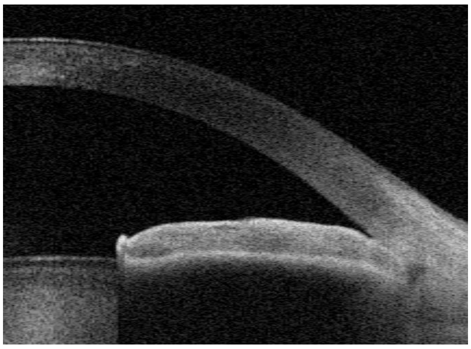

Figure 1 synechial angle closure

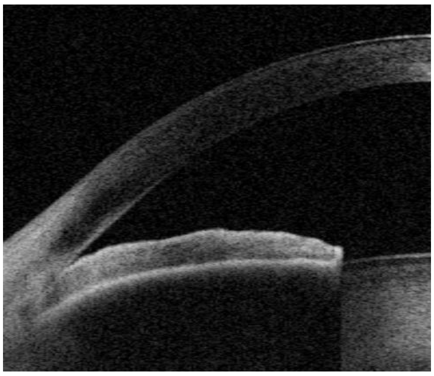

Figure 2 appositional angle closure

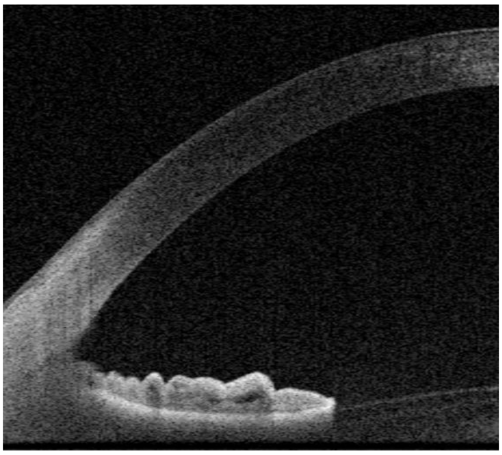

Figure 3 open angle

The raw data can't be freely available in the manuscript, the appendix, nor a public repository because of ethical restrictions. We have discussed about the possibility of data sharing with the Research Data Supervisory Committee of State Key Laboratory of Ophthalmology, Zhongshan Ophthalmic Centre, Sun Yat-sen University（SYSUZOC） who had exanimated and proved the authenticity of data. However the committee restricts the data from sharing. They suggest that more information about the data are available from SYSUZOC for researchers who meet the criteria for access to confidential data. Interested researchers can send data access requests to the corresponding author (yuminbin@mail.sysu.edu.cn/haot.lin@hotmail.com). So we attach 3 images of different classification to be presentation in supplementary.
