# Supplementary material for: Diagnostic Performance of Deep Learning Classifiers in Measuring Peripheral Anterior Synechia Based on Swept Source Optical Coherence Tomography Images
Source: Front Med (Lausanne). 2022 Jan 26;8:775711. doi: 10.3389/fmed.2021.775711 (PMC8825342; doi:10.3389/fmed.2021.775711)
Supplement: Supplementary file 3 [file Data_Sheet_2.PDF]

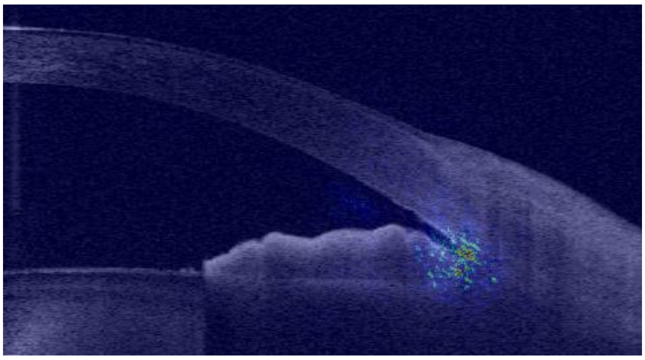

Figure 1 Heat map of open angle in the first deep learning classifier

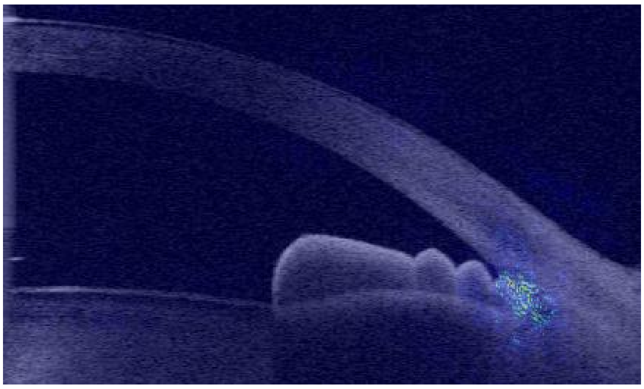

Figure 2 Heat map of static angle closure in the first deep learning classifier

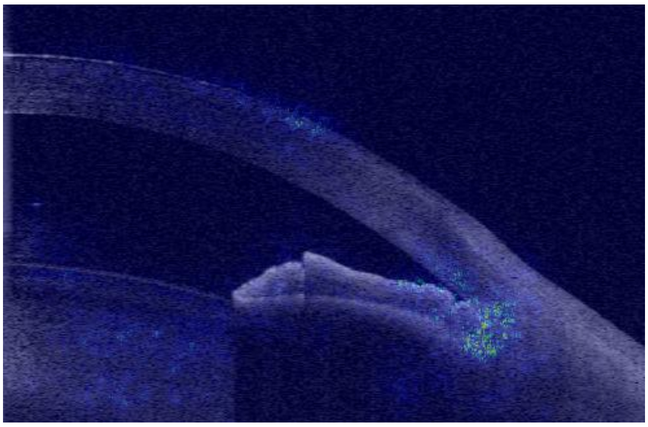

Figure 3 Heat map of appositional angle closure in the second deep learning classifier

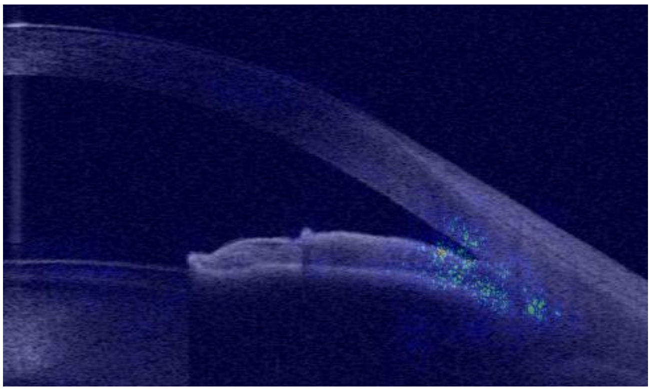

Figure 4 Heat map of synechial angle closure in the second deep learning classifier
